# The Clinical Efficacy of Accelerated Deep Repetitive Transcranial Magnetic Stimulation in Depression and Obsessive-Compulsive Disorder: Multi-centric Real-World Observational Data

**DOI:** 10.7759/cureus.60895

**Published:** 2024-05-23

**Authors:** Aswin K Mudunuru, M S Reddy, Kartik Valipay, Balaji S A, Madhiha M, Chandresh N, Chandrasekhar K, Prasad R Gundugurti

**Affiliations:** 1 Non-Invasive Brain Stimulation, Asha Neuromodulation Clinics, Hyderabad, IND; 2 Psychiatry, Asha Hospital, Hyderabad, IND; 3 Psychiatry, Asha Neuromodulation Clinic, Hyderabad, IND; 4 Psychiatry, Asha Neuromodulation Clinic, Bengaluru, IND; 5 Psychiatry and Behavioral Sciences, Asha Hospital, Hyderabad, IND

**Keywords:** depression, ocd, india, real-world, h-coil, deep tms

## Abstract

Background

Of late, the interest in accelerated treatment protocols in repetitive transcranial magnetic stimulation (TMS) for the treatment of depression and obsessive-compulsive disorder (OCD) has been gaining momentum. Studies have already found that the patterned theta burst stimulation is non-inferior to the standard high-frequency stimulation in treating depression. The objective of the present study was to evaluate the clinical efficacy of a customized accelerated combination TMS naturalistic setting.

Methods

Retrospective analysis of pre and post-deep repetitive TMS responses in depression and OCD patients was performed. About 391 Depression and 239 OCD patients' data was analyzed. Customized treatment protocols consisted of twice daily high-frequency stimulations intervened by one theta burst stimulation. The outcome measures were a day six score in depression and a day 10 score in OCD, compared to day one baseline scores.

Results

The overall response rate in depression was 60.86%, estimated as a >50% reduction in the Hamilton Depression Rating Scale (HAM-D) 21 items score, and 62.76% in OCD, estimated as a >35% reduction in the Yale-Brown Obsessive-Compulsive Scale (Y-BOCS) score. The mean reduction of YBOCS and HAM-D was statistically significant at p<0.0001 (Mann-Whitney U test statistic=9442.5, z=12.66 for YBOCS and 16673.5, z=18.92 for HAM-D). Corresponding effect size estimations revealed Cohen's d value of 1.40 and 1.59, respectively.

Conclusions

The response rates achieved at day six and day 10 in depression and OCD, respectively, were comparable to previous studies employing standard treatment protocols. The accelerated protocol produced satisfactory short-term clinical outcomes that were effective in the early management of the illness without any serious adverse effects.

## Introduction

Deep repetitive transcranial magnetic stimulation (dTMS) has been approved by the United States Food and Drug Administration (US-FDA) for its therapeutic application in major depressive disorder (MDD) in 2013 and in obsessive-compulsive disorder (OCD) in 2018 [1]. The H1 coil is known to stimulate the bilateral prefrontal cortex with more preference for the left dorsolateral prefrontal cortex (dlPFC) and is used in the treatment of MDD, whereas the H7 coil produces symmetric stimulation over bilateral dorsomedial prefrontal cortex (dmPFC) and anterior cingulate cortex (ACC) to treat OCD [2].

The therapeutic effects of transcranial magnetic stimulation (TMS) could be classified as classical and non-classical. Accordingly, modulation in neurotransmitter concentrations, synaptic plasticity via long-term potentiation, and long-term depression are the classical effects. TMS has also been shown to influence dendritic growth and sprouting through the production of neurotrophic factors like brain-derived neurotrophic factor [3]. The non-classical effects of TMS were related to the biophysical effects of magnetic fields, including the quantum effects, the magnetic spin effects, genetic magnetoreception, and macromolecular effects [3, 4]. A theoretical plausibility exists that these effects could be magnified by producing stronger and deeper TMS-induced electric fields (e-fields) in the brain to facilitate modulation of neuronal ensembles in a wide area as well as activation of projection neurons. Technologically, the H-coils produce broader and deeper e-fields up to a depth of about 3.5 cm from the cerebral cortex [5, 6]. This would somehow modulate, to a greater extent, the dysfunctional neural networks in mental illnesses like depression and OCD. In fact, there are multiple large-scale studies to support the point that the usage of dTMS as an adjuvant to pharmacotherapy has produced promising response and remission rates while following the standard treatment protocols [7-9]. Motor threshold (MT), session frequency, session number, and patterned stimulation are a few factors that majorly determine the clinical outcomes. For instance, an increase in session number improved the outcomes of OCD [10]. It is interesting to know that despite many factors affecting the treatment, there is still inter-individual and inter-session variability, as evidenced by many case reports and series. There are state and trait-dependent causes for such variability, as opined by a few authors [11]. State-dependent causes like psychiatric comorbidity, EEG-activity, cerebral metabolism, etc., and trait-dependent causes like personality types, anxiety, hormonal states, etc., could possibly affect this variability. We feel that a combination of TMS would help to minimize this variability. In India, multi-chain specialty clinics have been started in the private sector to offer dTMS treatment for depression and OCD from around April 2022. Accelerated and customized treatment protocols, with the primary intention of cutting short the duration of hospital stays or hospital visits for our patients, are being followed at these clinics. The idea of an accelerated TMS protocol and its efficacy has been studied previously [12]. Very recently, it has been opined that accelerated TMS appears to have the potential to hold promise and reduce treatment time in depression, concomitantly giving the desirable response [13]. We have been using the accelerated protocol comprising two sessions of high-frequency stimulation (HFS) interspersed with an intermittent theta burst stimulation (iTBS600). The HFS consisted of 18 Hz and 20 Hz in MDD and OCD, respectively.

The objective of the present study was to assess the efficacy of our accelerated protocol on the clinical outcomes in MDD and OCD, when the pharmacotherapy is maintained as usual. The outcome measures selected were day 6 Hamilton Depression Rating Scale with 21 items (HAM-D) and day 10 Yale-Brown Obsessive Compulsive Scale (Y-BOCS) for MDD and OCD respectively, as compared to their day one scores.

## Materials and methods

This retrospective observational study collected clinical outcomes of all the outpatients who visited the three neuromodulation clinics during the period April 2022 to December 2023 (21 months) using a consecutive sampling technique. The diagnosis was made using the Diagnostic and Statistical Manual of Mental Disorders, Fifth Edition (DSM-5) guidelines. The patient group was a mixed population with different drug prescriptions and durations of illness. All the patients have given written informed consent to use their treatment data for research purposes. Our study is a retrospective observational study of the clinical data, and ethical approval was not required. A psychiatrist screened the patients for electromagnetic field compatibility. All the patients diagnosed with MDD and OCD, with or without other medical comorbidities like hypertension, diabetes, etc., were included in the study. Patients belonging to both sexes and in the age group of 18-65 years were included. The presence of ferromagnetic materials in the head and neck region, a history of traumatic brain injury, and the presence of pacemakers, cochlear implants, or other implanted devices were the clear exclusion criteria [14, 15]. Patients with psychosis and severe substance use disorders were excluded. Clinical data of all the patients satisfying the inclusion criteria was considered for analysis. 

The primary outcome measures were the scores of the Hamilton Depression Rating Scale with 21 items (HAM-D) and Yale-Brown Obsessive-Compulsive Scale (Y-BOCS) measured after completion of 18 sessions (day six) for MDD and 30 sessions (day 10) for OCD, and compared to baseline day one score [16, 17]. The response rate in MDD was calculated as >50% reduction in HAM-D score on day six. Remission in MDD was considered at HAM-D <7 on day six. The response rate in OCD was calculated as >35% reduction in Y-BOCS score on day 10. As the guidelines demand the sustenance of response for at least one week post-treatment to be called as remission, we present only the proportion of patients whose Y-BOCS was <12 on day 10 and do not call this a remission rate. All rating scales were administered by psychologists before (day 1) and immediately after the treatment (day six or 10). The post-dTMS score was evaluated by another psychologist to rule out operator bias.

The device used was BrainsWay's 104 deep TMS system (BrainsWay, Jerusalem, Israel) fitted with H1 and H7 coils.

MDD treatment protocol followed every day for six days: first HFS session, followed by one iTBS600 session after one hour, further followed by a second HFS session after another hour. Each HFS session extended for about 20 minutes, consisting of 55 two-second trains with 18 Hz frequency and 20 seconds of interval given at 120% of RMT delivering 1980 pulses. An iTBS600 session consisted of 20 trains of frequency 5Hz, each consisting of 10 triplet bursts of 50 Hz, repeated with an interval of eight seconds. The total number of pulses delivered per day was 4560.

OCD treatment protocol followed every day for 10 days: same as above, but the HFS session consisted of 50 two-second trains with 20Hz frequency and 20 seconds of interval given at 100% of RMT, delivering 2000 pulses. The total number of pulses delivered per day, were 4600.

No specific behavioral techniques were employed during the session except supportive counseling.

Statistical analysis

The preliminary data was entered into Excel (Microsoft, Redmond, Washington), and statistical analysis was performed using SPSS software (IBM Inc., Armonk, New York). Table 1 shows the statistical tests used and the values obtained. A paired sample t-test was performed separately in men and women. The difference in mean reduction in HAM-D scores was highly statistically significant at p=0.0001 (t=16.524, r=0.698; t=20.312, r=0.658 in men and women respectively). Similarly, the difference in a mean reduction in Y-BOCS scores was also highly significant at p=0.0001 (t=12.857, r=0.803; t=11.488, r=0.803 in men and women, respectively). The non-parametric Mann-Whitney U test performed on the mean difference in Y-BOCS and HAM-D scores is also significant at p<0.001 (U=9442.5, z=12.66; U=16673.5, z=18.92 for ΔY-BOCS and ΔHAMD, respectively). Corresponding effect size estimations revealed Cohen's d value of 1.40 and 1.59, respectively.

**Table 1:** Statistical tests performed and the corresponding values and significance level HAM-D - Hamilton Depression Rating Scale; Y-BOCS - Yale-Brown Obsessive Compulsive Scale

Outcome measure	Statistical test performed	Group	Value of the statistic	Level of significance
Mean reduction in HAM-D score (day 1-6)	Paired t-test	Males	t=16.524; r=0.698	p=0.0001
		Females	t=20.312; r=0.658	p=0.0001
	Mann-Whitney U test	All patients	U=16673.5; z=18.92	p<0.001
Mean reduction in Y-BOCS score (day 1-10)	Paired t-test	Males	t=12.857; r=0.803	p=0.0001
		Females	t=11.488; r=0.803	p=0.0001
	Mann-Whitney U test	All patients	U=9442.5; z=12.66	p<0.001

## Results

The demographic data are presented in Table 2. Data of 391 MDD patients and 239 OCD patients was included for analysis. In either case, above 50% (N=429) of the patients presented with severe form of the disease (Table 3). All the patients were on pharmacotherapy, predominant drug being SSRI.

**Table 2:** Age and sex-wise distribution of patients in MDD and OCD groups OCD - obsessive-compulsive disorder; MDD - major depressive disorder

Diagnosis	Male + female		Only female		Only male	
	No. of patients (N)	Mean age (mean±SD)	No. of patients (N)	Mean age (mean±SD)	No. of patients (N)	Mean age (mean±SD)
OCD	239	34.08±12.38	116	35.53±13.04	123	32.72±11.55
MDD	391	37.98±14.66	212	36.12±14.36	179	40.20±14.70
Total	630		328		302	

**Table 3:** Disease severity distribution of OCD and MDD patients expressed as number (N) OCD - obsessive-compulsive disorder; MDD - major depressive disorder

Condition	Sex/severity	Mild (N)	Moderate (N)	Severe (N)	Extreme (N)	Total (N)
OCD	Female	13	25	48	30	116
	Male	17	28	46	32	123
	Total	30	53	94	62	239
MDD	Female	14	43	53	102	212
	Male	16	45	41	77	179
	Total	30	88	94	179	391

OCD results

**Figure 1:**
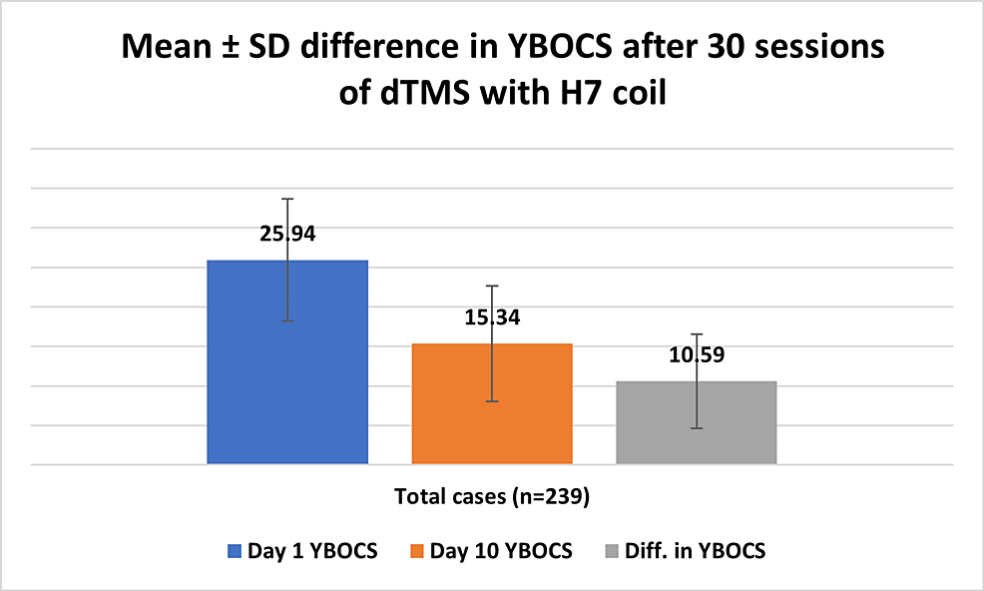
Reduction in Y-BOCS scores on day 10 expressed as mean±SD Y-BOCS - Yale-Brown Obsessive Compulsive Scale; dTMS - deep repetitive transcranial magnetic stimulation

The mean reduction in the absolute Y-BOCS scores on day 10 was 10.59±5.98 (Figure 1, Table 4). This score reduction was slightly higher in males 44.25±18.85% (N=123) as compared to 38.97±21.53% (N=116) in female patients (Table 4). Male patients with extreme OCD (N=32) showed a significantly better (p=0.2) improvement as compared to females with extreme OCD. The overall response rate was 62.76%. Also, 35.14% of the patients showed a day 10 Y-BOCS score of <12.

**Table 4:** Disease severity-wise and sex-wise mean reductions in Y-BOCS in OCD patients on day 10 expressed as mean±SD *statistically significant at p=0.0001 OCD - obsessive-compulsive disorder; Y-BOCS - Yale-Brown Obsessive Compulsive Scale

Measure	Y-BOCS range	Initial Y-BOCS score (Y1)		Final Y-BOCS score (Y2)		Diff. in Y-BOCS score *(Y2-Y1)		% Response	
		Mean	SD	Mean	SD	Mean	SD	Mean	SD
All patients (n=239)	Overall	25.94	7.75	15.34	7.33	10.59	5.98	41.69	20.36
	Mild	13.07	1.85	7.11	2.60	5.96	2.72	45.33	20.17
	Moderate	19.96	2.14	11.83	4.17	8.13	4.14	40.66	20.10
	Severe	27.37	2.25	15.48	5.64	11.89	5.59	43.46	20
	Extreme	35.39	2.31	22.34	6.60	13.05	6.88	36.67	18.65
Only male patients (n=123)	Overall	25.77	8.04	14.46	6.72	11.32	6.12	44.25	18.85
	Mild	12.65	2.93	6.65	2.70	6.0	2.52	49.44	20.28
	Moderate	19.64	2.16	11.75	4.15	7.89	4.03	40.13	19.57
	Severe	27.48	2.19	14.72	4.96	12.76	5.08	46.38	17.52
	Extreme	35.66	2.53	20.59	6.53	15.06	6.85	42.03	18.17
Only female patients (n=116)	Overall	26.11	7.44	16.28	7.81	9.83	5.73	38.97	21.53
	Mild	12.23	2.83	6.69	3.36	5.53	2.89	46.83	25.22
	Moderate	20.32	2.05	11.92	4.18	8.4	4.25	41.25	20.66
	Severe	27.27	2.30	16.21	6.13	11.06	5.92	40.67	21.75
	Extreme	35.10	2.00	24.20	6.15	10.9	6.22	30.94	17.41

MDD results

**Figure 2:**
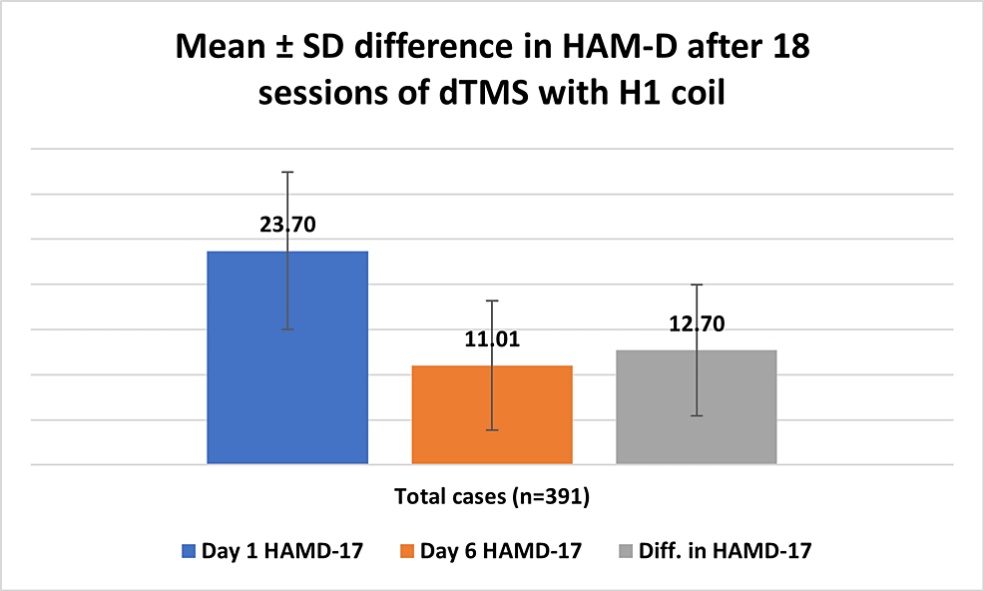
Reduction in HAM-D scores on day six expressed as mean±SD HAM-D - Hamilton Depression Rating Scale; dTMS - deep repetitive transcranial magnetic stimulation

The mean reduction in the absolute HAM-D scores on day six was 12.70±7.27 (Figure 2, Table 5). This reduction was higher in females 56.39±21.82% (N=212) as compared to 50.60±21.65% (N=179) in male patients (Table 5). In the severe depression group, there was no significant sex-wise difference. The overall response rate was 60.86%, and the remission rate was 25.31%.

**Table 5:** Disease severity and sex-wise mean reductions in HAM-D in MDD patients on day six expressed as mean±SD * statistically significant at p=0.0001 HAM-D - Hamilton Depression Rating Scale; MDD - major depressive disorder

Measure	HAMD range	Initial HAMD score (D1)		Final HAMD score (D2)		Diff. in HAMD score *(D2-D1)		% Response	
		Mean	SD	Mean	SD	Mean	SD	Mean	SD
All patients (n=391)	Overall	23.70	8.73	11.0	7.14	12.70	7.27	53.74	21.93
	Mild	11.22	1.57	4.85	2.51	6.37	3.16	55.34	25.03
	Moderate	16.32	1.40	7.04	3.56	9.27	3.49	56.98	21.12
	Severe	20.58	1.18	10.27	4.31	10.31	4.33	50.05	20.95
	Very severe	31.16	6.91	14.37	8.28	16.78	8.02	54.36	21.74
Only male patients (n=179)	Overall	22.85	8.40	11.18	6.59	11.67	6.82	50.60	21.65
	Mild	9.81	2.67	5.13	3.0	4.69	3.60	44.95	29.89
	Moderate	16.33	1.41	7.60	3.30	8.73	3.40	53.38	20.11
	Severe	20.66	1.14	11.07	4.47	9.59	4.79	46.04	22.06
	Very severe	30.53	6.23	14.58	7.45	15.95	7.12	52.58	19.56
Only female patients (n=212)	Overall	24.42	8.95	10.86	7.57	13.57	7.53	56.39	21.82
	Mild	11.57	1.49	4.42	1.63	7.14	2.64	60.12	18.16
	Moderate	16.30	1.39	6.46	3.74	9.87	3.50	60.76	21.5
	Severe	20.53	1.22	9.66	4.07	10.87	3.84	53.16	19.5
	Very severe	31.64	7.36	14.22	8.86	17.42	8.58	55.71	23.16

There were no seizures or other serious adverse events reported. 8.9% (N=56) of all the patients had experienced mild headache or application site pain which was self-limiting. There was no report on treatment-emergent manic switch in any of the patients (Table 6).

**Table 6:** Side-effects observed in patients who underwent accelerated dTMS treatment OCD - obsessive-compulsive disorder; MDD - major depressive disorder; dTMS - deep repetitive transcranial magnetic stimulation

Diagnosis	Sex	Side-effect (number of patients (% of total))					Total
		Facial twitches	Headache-mild/ pain in the application site	Headache-severe	Heaviness of head	None	
MDD	Male	0	16	1	0	162	179
	Female	1	11	2	1	197	212
OCD	Male	1	14	1	1	106	123
	Female	2	15	1	1	97	116
	Total	4 (0.6)	56 (8.9)	5 (0.8)	3 (0.4)	562 (89)	630 (100)

## Discussion

This real-world study performed in a naturalistic setting on a large sample is the first of its kind in India. The accelerated treatment protocol that we developed did prove to be reasonably effective in giving good clinical outcomes in MDD and OCD patients. We followed certain guidelines in designing the accelerated protocol, keeping in mind the gap between the successive sessions and the addition of an iTBS600 stimulation [18]. The inclusion of an iTBS session between two HFS sessions was the core concept of our protocol. A lot of review work has been done to decide on the inclusion of an iTBS session. Theta burst stimulation is known to promote long-term potentiation individually, similar to an HFS [19, 20]. However, there are mixed opinions on the pulse number in an iTBS session. While a few studies suggest 600 pulses to be beneficial, few case reports have shown effectiveness with an extended number of pulses [21]. Moreover, the clinical efficacy of the iTBS600 and the standard HFS for depression have been found to be similar in previous studies [22]. Therefore, we believe that iTBS600 would be the most suitable session to pad between two successive HFS sessions. Although the clinical effects of accelerated TMS have been documented earlier, clinically employing a tailor-made treatment protocol in a large number of patients has been taken up for the first time, to the best of our knowledge. As mentioned before, our primary motive behind customization was to minimize the hospital visits by the patient.

In the case of MDD protocol, a total of 27,360 pulses were administered in contrast to 39,600 delivered in 20 sessions as per standard HFS sessions advocated by BrainsWay [23]. Similarly, the OCD patients received a total of 46,000 pulses against the standard of 60,000. It is evident through our observational study that the number of sessions but not the dosage are important in producing a clinical outcome with dTMS. A special note must be made about the nil adverse events in all the 630 patients. Except for a self-limiting mild headache, there were no serious side effects during the treatment (Table 5). Our internal feedback from the patients showed the overall experience with dTMS treatment to be satisfactory.

Despite the study group heterogeneity and non-uniformity in drug prescriptions, our study has clearly shown that dTMS plays a key role in lowering the severity of the disease within the first week of treatment. The response rates estimated in our study were comparable to other clinical trials performed by the BrainsWay as well as individual authors.

The current retrospective study has four limitations. Firstly, the absence of a control group is the biggest limitation. Secondly, non-uniformity of the sample with regard to medication history and duration of illness. Although the medication history or duration of illness are non-uniform, they are, at the same time, not too different so as to pose a serious confounding effect. Thirdly, the rating scales were scored on day six and day 10 after the initiation of treatment, unlike the standard one-week duration. Lastly, the sustainability of the response could not be ascertained as the follow-up scores were not taken.

The implications of the study are that accelerated dTMS treatment proved effective despite the study population being heterogeneous and an early response (day six in MDD and day 10 in OCD) is quite possible with the same. 

The present study has the following recommendations: a) Abbreviated session number is equally effective to the extended sessions in producing the short-term response in MDD and OCD patients; b) Replacement of high-frequency sessions with an iTBS did not affect the outcome. This may be further studied by using exclusive accelerated iTBS sessions; c) The early onset of the effect is clinically promising, but the persistence of the effect must be estimated through follow-up studies; d) Accelerated protocol may not be reserved only for treatment-resistant cases but can be taken up in early course management of MDD and OCD.

Future directions

The authors believe that accelerated protocols are the need of the hour, and they could be replicated in multiple other centres. Neurophysiological monitoring and evaluation of the patient during the dTMS treatment would throw some light into understanding the mechanisms behind this clinical outcome.

## Conclusions

The modified accelerated protocol comprising two HFS and one iTBS600 stimulation produced good short-term clinical outcomes on day six in MDD and day 10 in OCD. This helps the clinicians with better early management of the illness. This could especially be useful in certain patient populations who are otherwise insensitive to longer treatment periods. The treatment did not produce significant adverse effects, proving its safety and acceptance. A distinctive early response is obtained with the accelerated protocol, which will help the clinician with the early management of MDD or OCD. More studies in this line are required to establish the clinical non-inferiority of accelerated protocols compared to standard regimens.
